# The psychological and political correlates of conspiracy theory beliefs

**DOI:** 10.1038/s41598-022-25617-0

**Published:** 2022-12-15

**Authors:** Joseph Uscinski, Adam Enders, Amanda Diekman, John Funchion, Casey Klofstad, Sandra Kuebler, Manohar Murthi, Kamal Premaratne, Michelle Seelig, Daniel Verdear, Stefan Wuchty

**Affiliations:** 1 Department of Political Science, University of Miami, 1300 Campo Sano Blvd., Coral Gables, FL 33146 USA; 2 Department of Political Science, University of Louisville, Louisville, KY 40292 USA; 3 Department of Psychology, Indiana University, Bloomington, IN 47405 USA; 4 Department of English, University of Miami, Coral Gables, FL 33146 USA; 5 Department of Linguistics, Indiana University, Bloomington, IN 47405 USA; 6 Department of Electrical and Computer Engineering, University of Miami, Coral Gables, FL 33146 USA; 7 Department of Cinema and Interactive Media, University of Miami, Coral Gables, FL 33146 USA; 8 Department of Computer Science, University of Miami, Coral Gables, FL 33146 USA

**Keywords:** Human behaviour, Psychology

## Abstract

Understanding the individual-level characteristics associated with conspiracy theory beliefs is vital to addressing and combatting those beliefs. While researchers have identified numerous psychological and political characteristics associated with conspiracy theory beliefs, the generalizability of those findings is uncertain because they are typically drawn from studies of only a few conspiracy theories. Here, we employ a national survey of 2021 U.S. adults that asks about 15 psychological and political characteristics as well as beliefs in 39 different conspiracy theories. Across 585 relationships examined within both bivariate (correlations) and multivariate (regression) frameworks, we find that psychological traits (e.g., dark triad) and non-partisan/ideological political worldviews (e.g., populism, support for violence) are most strongly related to individual conspiracy theory beliefs, regardless of the belief under consideration, while other previously identified correlates (e.g., partisanship, ideological extremity) are inconsistently related. We also find that the correlates of specific conspiracy theory beliefs mirror those of *conspiracy thinking* (the predisposition), indicating that this predisposition operates like an ‘average’ of individual conspiracy theory beliefs. Overall, our findings detail the psychological and political traits of the individuals most drawn to conspiracy theories and have important implications for scholars and practitioners seeking to prevent or reduce the impact of conspiracy theories.

## Introduction

Conspiracy theory beliefs are associated with numerous societal harms, including vaccine refusal, prejudice against vulnerable groups, and political violence^1–6^. To lay the groundwork for the development of effective and practical tools to minimize such harms, broad, interdisciplinary research programs have developed over the past decade^7–11^. The growing literature has collectively identified dozens of individual-level psychological and political factors that are correlated with conspiracy theory beliefs^11^. However, the literature has developed in a piecemeal fashion, with singular studies oftentimes considering only a small number of conspiracy theories or potential correlates at a time^12^. This brings into question the generalizability of these previous findings.

Our central concern is the extent to which the previously identified psychological and political correlates of conspiracy theory beliefs vary—in strength, direction, and statistical significance—depending on the specific conspiracy theory belief being examined. For example, Republicanism and conservatism are typically associated with the belief that Barack Obama faked his birth certificate^13^. Such a finding reveals important details about the basic nature of “birther” beliefs and could even be used to develop strategies to correct such beliefs^14^. But should we also expect that the factors related to birtherism are also related to beliefs in other conspiracy theories, such as the assertion that the moon landing was faked? Are the characteristics related to birtherism similar to those of the average conspiracy theory believer, or specific to believers in birtherism or a few other conspiracy theories? Similar questions may be asked of the political and psychological characteristics associated with believers of other conspiracy theories. Going further, should we expect because, for example, narcissism is associated with Holocaust denial and support for violence with QAnon beliefs that, on average, those exhibiting a tendency toward generalized conspiracy thinking are also likely to be narcissistic or supportive of violence? In each case the literature only offers speculation because generalizability is rarely considered.

Our research question asks: to what extent do the major psychological and political correlates of conspiracy theory beliefs fluctuate depending on the specific conspiracy theory belief under consideration? To answer, we aim to construct empirical distributions of the effects of various political and psychological characteristics on a wide variety of conspiracy theory beliefs. The distribution of these effects provides clues as to how common (or uncommon) a relationship between a given conspiracy theory belief and political/psychological characteristic is, thereby shedding light on the characteristics of the average conspiracy believer and potentially qualifying past inferences made using beliefs in only a single or small number of conspiracy theories. In short, our analysis is calibrated to decipher how generalizable and representative previously identified characteristics of conspiracy believers really are.

To this end, we employ a national survey of 2021 U.S. adults from May 2021, estimating previously identified relationships across many conspiracy theory beliefs within both bivariate (correlation) and multivariate (regression with controls) frameworks. In particular, we focus on 15 different individual-level psychological and political characteristics identified by past work and beliefs in 39 conspiracy theories that vary by topic, the supposed villains, the age of the theory, and its popularity—585 pairwise relationships in total. We also consider the relationships between the 15 individual-level characteristics and *conspiracy thinking*, the predisposition toward viewing events and circumstances in conspiratorial terms^15–17^, which allows for a comparison of these relationships with those we observe with beliefs in specific conspiracy theories. Of course, we should hope that patterns match, but this is ultimately an empirical question that remains unanswered by the literature. Should the relationships between the various correlates we consider and the specific conspiracy theory beliefs we employ closely match the relationships between those correlates and the conspiracy thinking predisposition, there would be good empirical reason for researchers—in many research designs—to avoid the trappings of specific conspiracy theories in favor of analyzing the general predisposition.

The vast majority of research on the political correlates of conspiracy theory beliefs focuses on partisanship and ideology^16, 18–22^. Some studies distinguish between partisanship and ideology and the strength of those attachments, finding that—regardless of partisan/ideological “direction”—the extremity of one’s political attachments are associated with conspiracy beliefs^21, 22^. After Donald Trump was elected, more research considered the role of orientations toward Trump^23^, specifically, as well as other orientations that have been associated with Trump, such as populism and Manicheanism^24–32^. Finally, support for political violence has become relevant in the wake of the January 6, 2021 riot^33^, the burning of 5G cell tower^3^, and other acts of violence during the COVID pandemic and 2020 election^34^. Altogether, we believe that we have broad coverage of the political correlates of conspiracy beliefs identified in past literature.

Our examination of psychological correlates is primarily confined to personality traits, such as Machiavellianism, psychopathy, and narcissism—each of which are related to conspiracy theory beliefs^35–37^. We also consider potential downstream products of these antisocial personality traits that have also been found to correlate with conspiracy theory beliefs, such as the tendency to knowingly share false information online, support the use of violence, and distrust government and other people^35, 38–40^. We focus on personality traits because they—perhaps unlike other psychological factors such as cognitive biases (e.g., conjunction fallacy, intentionality bias) or existential motives (e.g., feelings of powerlessness)—appear to actually structure conspiratorial belief systems^38^. This is not to say that cognitive biases and existential motives of various sorts are not fundamental or important, but they appear to be less predictive of conspiracism than personality traits and other political orientations^41^. Although the list of correlates we consider is necessarily incomplete, it does broadly cover the political and social-psychological correlates identified by past literature as being strongly predictive of a wide variety of beliefs in specific conspiracy theories.

The answer to our primary research question—to what extent do the major psychological and political correlates of conspiracy theory beliefs fluctuate depending on the specific conspiracy theory belief under consideration—has important implications for the study of conspiracy theories. First, it may call into question the generalizability of inferences made using a small number of conspiracy theory beliefs (which is the strategy employed by most studies), especially when the conspiracy theories examined in singular studies exhibit similar characteristics (e.g., who the accused conspirators are). Second, our findings may help with the development of strategies designed to curtail conspiracy theory beliefs and their associated harms. The development of effective interventions requires an accurate understanding of the correlates of conspiracy theory beliefs, as these can shed light on believers’ motivations and tendencies.

## Materials and methods

### Survey

We surveyed U.S. adults in partnership with Qualtrics between April 30–May 19, 2021. The University of Miami Human Subjects Research Office approved this survey on April 8, 2021 (#20210244). Survey respondents provided informed consent via computer screen and could exit at any time. This research was conducted in accordance with all relevant guidelines and regulations, including the Declaration of Helsinki. All materials necessary for replication are available in the Open Science Framework. The quota-based sampling procedure produced a sample representative of the American adult population in terms of gender, age, race and ethnicity, household income, and educational attainment based on 2019 American Community Survey estimates; see the supplementary information for details about the precise sociodemographic composition of the sample.

We took several steps to ensure response quality. Following best practices^42^, our survey included four attention checks (two standalone and two embedded in grids of Likert response-type questions). Respondents who did not successfully complete all four attention checks were excluded from the sample. We also excluded “speeders”: respondents who took less than half the median time to complete the survey upon “soft launch” of the survey on a limited sample. The final sample size was 2021 U.S. adults.

### Dependent variables

We employ two sets of dependent variables: the first capturing beliefs in 39 specific conspiracy theories and the second measuring conspiracy thinking (the predisposition towards believing conspiracy theories, sometimes called “conspiracy mentality" or “conspiracy ideation")^43^. Regarding our specific conspiracy theories, we note that researchers cannot examine *every* conspiracy theory because the universe of conspiracy theories is constantly expanding and seemingly infinite^44^. Consequently, an investigation of beliefs in *all* or even *most* conspiracy theories is impossible. Moreover, researchers have yet to identify the “right” set of conspiracy theories for the purpose of making generalizable inferences, prompting the need for the research at hand^45^.

We therefore examine a large number of conspiracy beliefs—39 in total—that capture the five types of conspiracy theories identified by Brotherton, French, and Pickering^46^: government malfeasance (e.g., government officials engaged in sex trafficking), extraterrestrial cover-up (e.g., government hiding alien contact), malevolent global conspiracies (e.g., The Rothschilds control the world), personal well-being (e.g., 5G cell towers spread coronavirus), and control of information (e.g., FDA hiding cures). As many partisan conspiracy theories are popular in the U.S., we also include conspiracy theories involving partisan actors and issues (e.g., Hillary Clinton gave Russia nuclear materials).

The supplementary information lists the 39 items, as well as the percentage of Americans who express belief in each. These conspiracy theories vary considerably in their popularity, ranging from a low of 5% (Osama bin Laden is still alive) to a high of 56% (more than one person was behind the assassination of President Kennedy).

We measure the predisposition to interpret events and circumstances as the product of real-world conspiracies—*conspiracy thinking*—using the American Conspiracy Thinking Scale (ACTS). The ACTS is an index of four questions—each measured on a five-point, “strongly disagree” (1) to “strongly agree” (5) scales—developed by Uscinski and Parent^47^ and based on items employed by McClosky and Chong^48^:

1. Much of our lives are being controlled by plots hatched in secret places.
2. Even though we live in a democracy, a few people will always run things anyway.
3. The people who really “run” the country are not known to the voters.
4. Big events like wars, the current recession, and the outcomes of elections are controlled by small groups of people who are working in secret against the rest of us.

This scale (range = 1–5, *M* = 3.11, *SD* = 1.00, α = 0.86) has been validated in previous work^15–17, 30, 49–52^, which reveals correlations with a variety of conspiracy theory beliefs, as well as its reliable and unidimensional nature.

### Independent variables

We employ three substantive groups of independent variables—psychological and personality characteristics, non-partisan/ideological political attitudes, and partisan/ideological political attitudes and identities—comprised of 15 distinct variables in total. As for psychological and personality characteristics, we examine the relationship between conspiracy theory beliefs and the dark triad^53^, which includes narcissism (range = 1–5, *M* = 2.42, *SD* = 0.98, α = 0.87; e.g., “I tend to want others to pay attention to me”), Machiavellianism (range = 1–5, *M* = 2.09, *SD* = 0.91, α = 0.84; e.g., “I tend to manipulate others to get my way”), and psychopathy (range = 1–5, *M* = 2.12, *SD* = 0.87, α = 0.81; e.g., “I tend to be callous or insensitive”). Previous research has found that the dark triad is related to conspiracy theory beliefs that revolve around the Holocaust^38^, false flag events^38^, election fraud^54^, and QAnon^55^. We also consider the tendency to share false information online (range = 1–5, *M* = 1.81, *SD* = 1.09; e.g., “I share information on social media about politics even though I believe it may be false”), which is related to conspiracy theory beliefs regarding AIDS, school shootings, and COVID-19, for example^38^.

As for non-partisan/ideological political attitudes, we consider Manicheanism (range = 1–5, *M* = 3.25, *SD* = 1.17; e.g., “Politics is a battle between good and evil”), populism (range = 1–5, *M* = 3.80, *SD* = 0.81, α = 0.82; e.g., “The established elite and politicians have often betrayed the people”), support for political violence (range = 1–5, *M* = 2.16, *SD* = 1.24; e.g., “Violence is sometimes an acceptable way for Americans to express their disagreement with the government”), and (dis)trust in government (range = 1–5, *M* = 2.62, *SD* = 1.14; e.g., “The federal government in Washington can be trusted to do what is right”). Previous studies find that Manichean attitudes are related to several conspiracy theory beliefs^56^, as are populist attitudes and support for violence^26, 33, 55^. A long line of literature also theorizes and observes correlations between distrust and conspiracy theory beliefs^11^.

Finally, partisan/ideological attitudes include partisan (range 1–7, *M* = 3.73, *SD* = 2.20; 7 = “strong Republican”; e.g., “Generally speaking, do you usually think of yourself as a Republican, a Democrat, an Independent, or something else?”) and ideological (range = 1–7, *M* = 4.04, *SD* = 1.80; 7 = “extremely conservative”; e.g., “Where would you place yourself on a scale that goes from ‘very liberal’ to ‘very conservative’?”) identities, as well as the extremity of partisan (range = 1–4, *M* = 1.91, *SD* = 1.11; 4 = “strong partisan”) and ideological (range = 1–4, *M* = 1.37, *SD* = 1.17; 4 = “extreme identifier”) identities. Both strength of identity measures are “folded” versions of the seven-point partisan and ideological identity measures—the direction of attachment is removed and only strength/extremity of attachment remains. Previous studies show that partisan and ideological identities are inconsistently associated with conspiracy theory beliefs^20, 57^: some conspiracy theories are believed more by those on the political right^19, 58^, and others find more support among those on the left^56^. Furthermore, some studies find that conspiracy theory beliefs are related to partisan strength and ideological extremity^22^, while others find that conspiracy theory beliefs are unrelated to partisanship, ideology or to the strength/extremity thereof^17^.

We also employ feelings toward salient partisan figures in the U.S. such as current President Joe Biden (range = 0–100, *M* = 50.51, *SD* = 38.12; this is assessed vis-à-vis a “feeling thermometer” where 100 = very positive feelings) and former President Donald Trump (range = 0–100, *M* = 39.70, *SD* = 39.15; this is assessed vis-à-vis a “feeling thermometer” where 100 = very positive feelings). Support for Donald Trump is correlated with beliefs in conspiracy theories regarding COVID-19^23^, election fraud^59^, and QAnon^55^, for example.

### Methods

Our analysis unfolds in two steps. First, we estimate the correlation between each of the 15 characteristics described above and each of the 39 conspiracy theory beliefs, for a total of 585 correlations (product-moment correlations for conspiracy theory questions that utilize ordinal response options and point-biserial correlations for those that employ dichotomous response options; see supplementary information for details). We examine distributions of these correlations by characteristic, paying particular attention to the mean and standard deviation, as well as the proportion of instances in which the correlations are statistically distinguishable from 0 at *p* < 0.05 (using Benjamini–Hochberg to estimate the false discovery rate across many tests). By examining distributions of correlations across a wide variety of conspiracy theory beliefs, we can better understand the extent to which such correlations fluctuate in magnitude, direction, and statistical significance as the details of the specific conspiracy theories in question vary. We also bolster this analysis by examining the distribution of coefficients from regressions of each conspiracy belief on the full set of individual-level characteristics.

Second, we examine correlations between each of the 15 identified correlates and our measure of generalized conspiracy thinking, the ACTS. We pay particular attention to the extent to which these patterns reflect those in our first analysis: do the correlations with the general predisposition tend to mimic the average correlations across a wide range of specific beliefs?

### Ethics

Survey protocol was approved by the University of Miami Institutional Review Board (Protocol ##20210244). Survey respondents provided informed consent and could leave the survey at any time. All relevant regulations were followed in performing this research.

## Results

**Figure 1:**
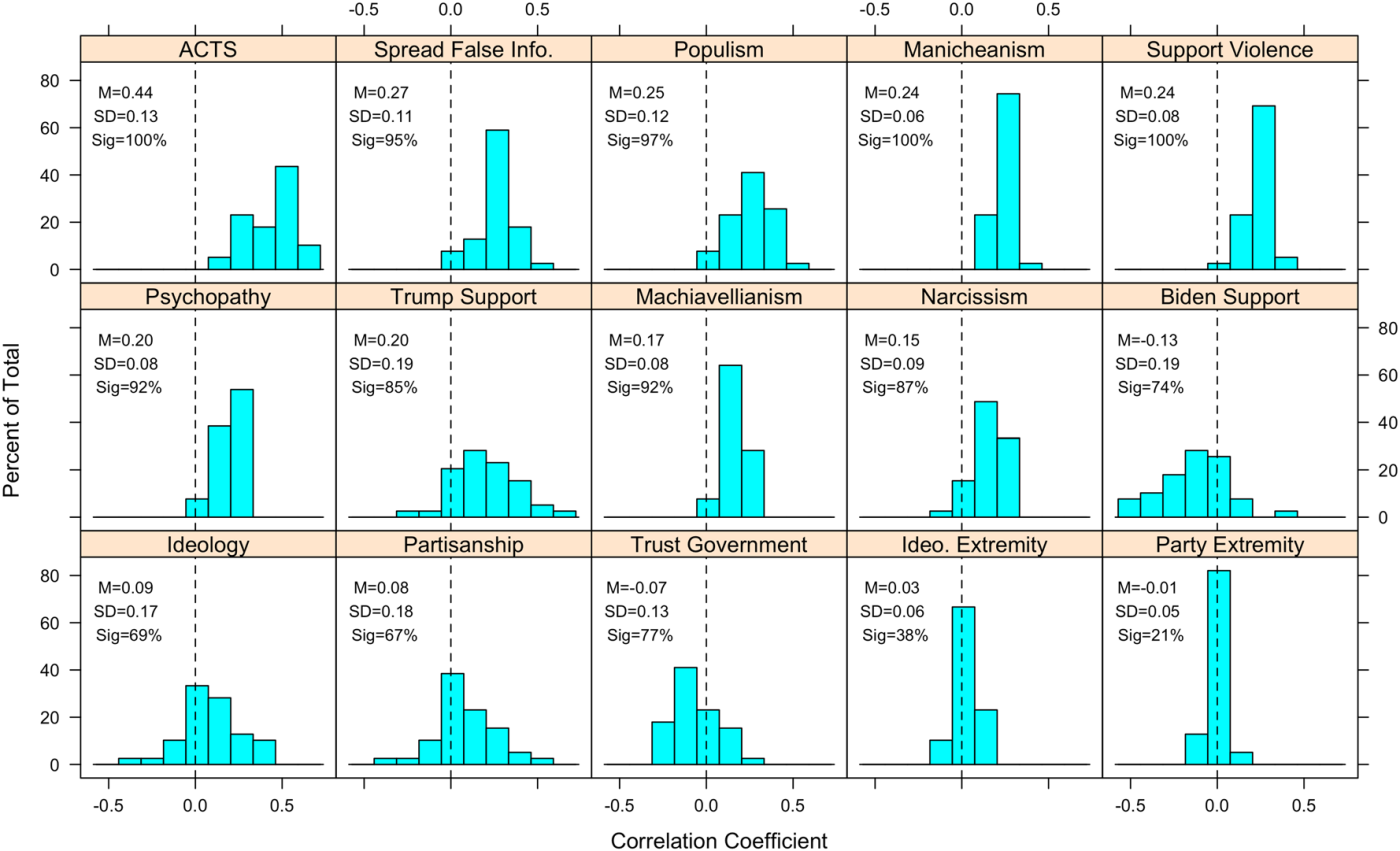
Distribution of correlation coefficients, by psychological and political correlates, across all conspiracy theory beliefs. Mean, standard deviation, and percentage of cases where correlation was statistically significant (*p* < 0.05) appears in text. *p*-values corrected for multiple comparisons via the Benjamini–Hochberg procedure.

Figure 1 displays the distribution of correlations between each of the 15 characteristics and the 39 conspiracy theory beliefs we employ. In each panel, we include the mean and standard deviation of the distribution, as well as the percentage of the 39 cases where the correlation is statistically distinguishable from 0 at *p* < 0.05. Several patterns emerge.

First, the average correlation with our measure of conspiracy thinking (the ACTS), 0.44, is nearly double that of the next largest average correlation, 0.27, which is associated with spreading false information online (though this correlation is significant across 95% of conspiracy theory beliefs, compared to 100% for the ACTS). This makes theoretical sense and showcases the validity and practical utility of measures of conspiracy thinking such as the ACTS. For the dark triad traits, we observe distributions of mostly positive correlations with small standard deviations; Machiavellianism, narcissism, and psychopathy, are statistically significant correlates of 92%, 87%, and 92% of our conspiracy theory beliefs, respectively.

Results are similar for non-partisan/ideological political attitudes, particularly populism, Manicheanism, and support for political violence. These attitudes are significant in 97–100% of cases with relatively strong mean correlations ranging from 0.24 to 0.25. The exception is trust in government, which we expect to be negatively correlated with conspiracy theory beliefs. While the distribution is skewed such that there are more negative correlations than positive ones, it is also centered near 0, with a mean correlation of − 0.07 and standard deviation larger than the other correlates we have discussed so far (0.13). Moreover, trust in government has a lower significance rate of 77%.

Finally, we consider partisan/ideological attitudes and identities. We observe very similar patterns when it comes to partisanship and ideology: relatively weak average correlations of 0.08–0.09, large standard deviations of 0.17–0.18 relative to the other correlates, and a mixture of positive and negative correlations. Both identities are significantly correlated with 69% of the conspiracy theory beliefs we consider (i.e., 26–27 beliefs). This comports with recent work showing inconsistent correlations between partisan and ideological identities depending on the details of the conspiracy theory in question, namely the centrality of partisan/ideological figures and groups^20, 56^. A similar inference can be made about support for Joe Biden and Donald Trump, though feelings about these salient leaders are more strongly and consistently related than partisan and ideological identities. Both show average correlations greater than 0 in absolute value (0.20 for Trump, − 0.13 for Biden), with relatively large standard deviations, a mixture of positive and negative correlations across beliefs, and significance rates of 74% (Biden) and 85% (Trump).

We find the weakest evidence for a consistent effect of partisan and ideological extremity. Both have distributions with very small standard deviations and correlations clustered around 0; the average correlation is − 0.01 for partisan extremity, 0.04 for ideological extremity. Moreover, neither characteristic is significantly correlated in a majority (> 50%) of cases.

In the supplementary information, we replicate Fig. 1 separately for conspiracy theory questions that utilize ordinal response options and dichotomous response options (not including “don’t know” responses) in order to decipher whether the patterns observed above are impacted by question format. Although the point-biserial correlations utilized with the dichotomous items are weaker than the product-moment correlations (as to be expected), patterns are very similar. We also observe slightly greater average correlations for partisan/ideological political predispositions when it comes to the ordinal response items, though this appears to be an artifact of the fact that more of these items involve partisan or ideological groups, figures, and topics.

**Figure 2:**
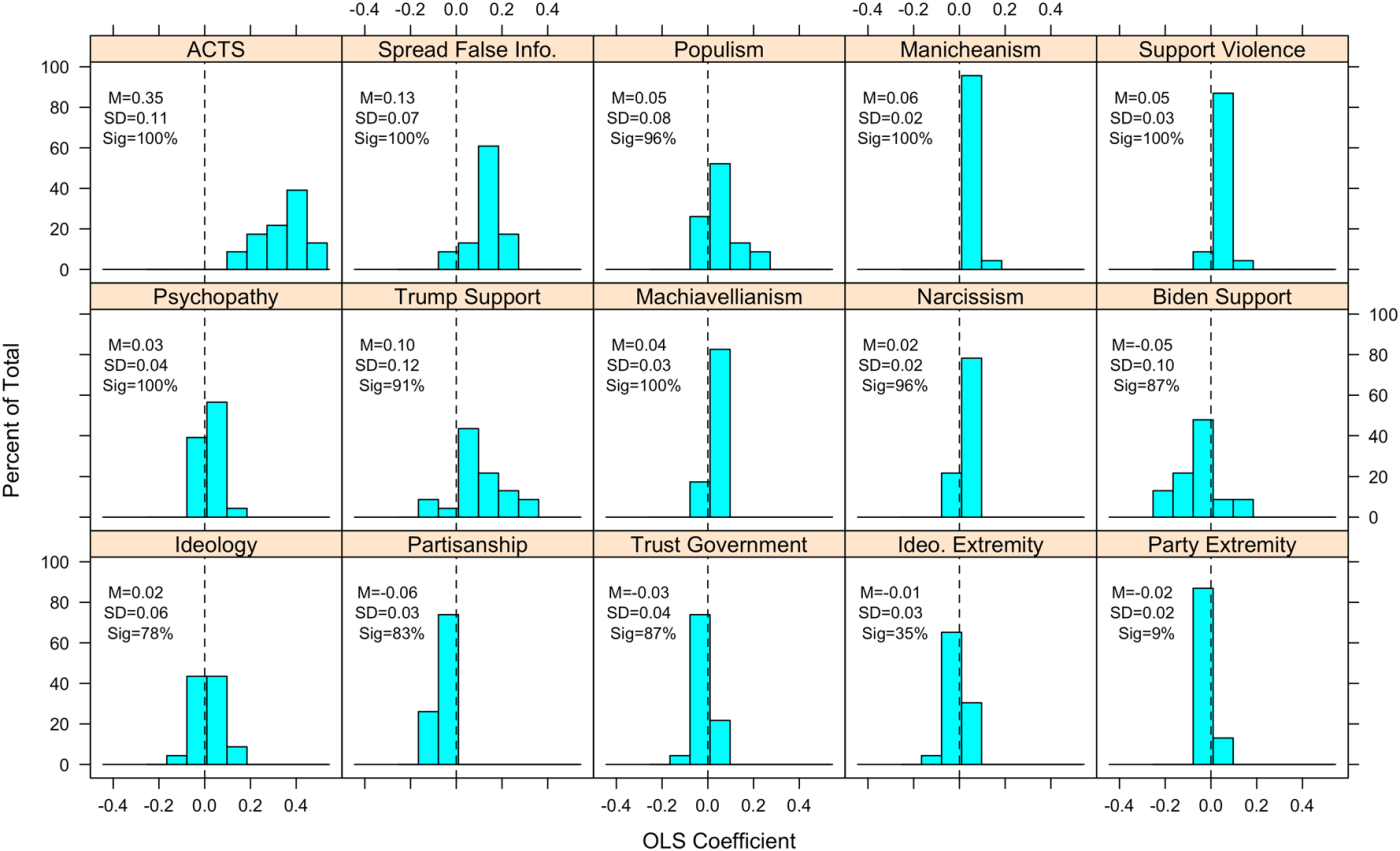
Distribution of standardized OLS coefficients for ordinal conspiracy belief questions, by psychological and political correlates. Mean, standard deviation, and percentage of cases where coefficient was statistically significant (*p* < 0.05) appears in text. *p*-values corrected for multiple comparisons via Benjamini–Hochberg procedure.

**Figure 3:**
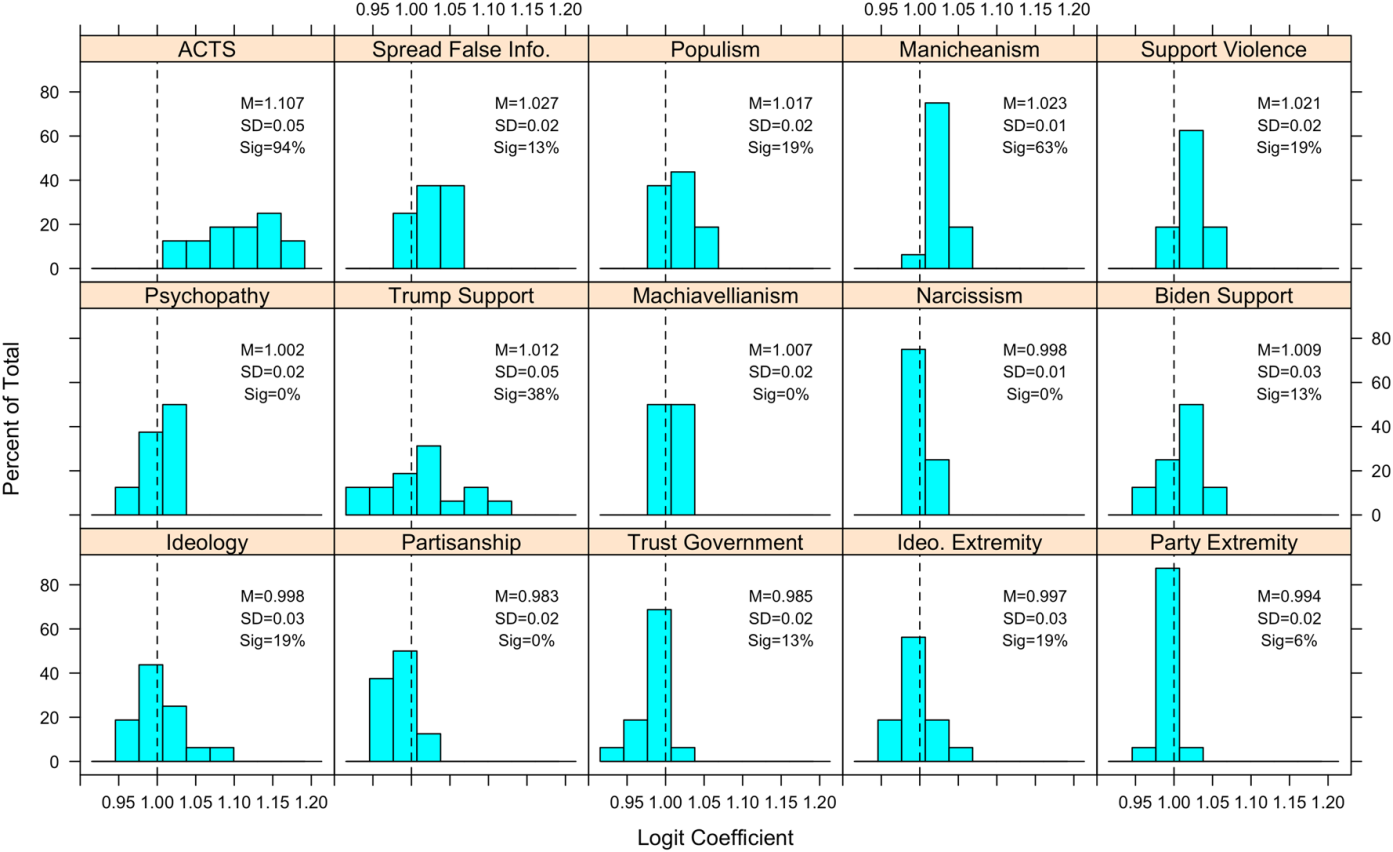
Distribution of odd ratios (based on logit coefficients) for dichotomous conspiracy belief questions, by psychological and political correlates. Mean, standard deviation, and percentage of cases where coefficient was statistically significant (*p* < 0.05) appears in text. *p*-values corrected for multiple comparisons via Benjamini–Hochberg procedure.

Next, we replicate Fig. 1 in a regression framework whereby each conspiracy belief is regressed (via OLS or logistic regression) on all 15 predictors in order to examine controlled relationships. Results appear in Figs. 2 and 3. The ACTS is the strongest and most consistently significant predictor. Partisanship, ideology, and the strength thereof tend to be weak and non-significant on balance, though Trump support exhibits a more consistently moderate-strong relationship. We also find that many of the psychological and non-partisan/ideological (e.g., populism, support for political violence) predictors tend to exhibit weaker relationships than the correlational analysis in Fig. 1 revealed—this is likely because many of these variables are quite highly correlated with each other, and with ACTS (see supplementary information for these correlations). In other words, not only are inferences tethered to which conspiracy theories one utilizes, they are also contingent on which factors (if any) are controlled for in analyses.

**Figure 4:**
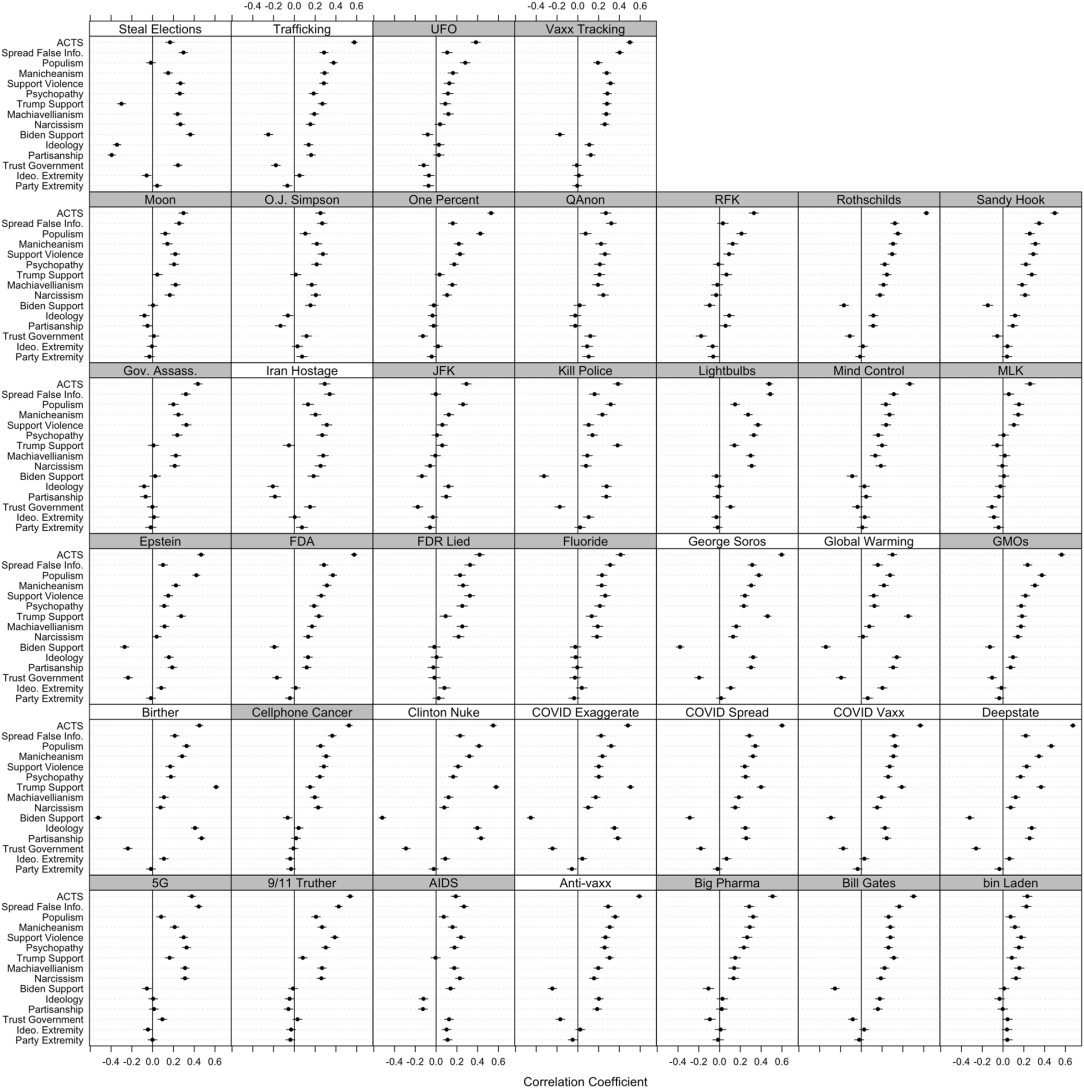
Pearson correlations between each psychological and political characteristic and each of 39 conspiracy theory beliefs. Partisan/ideological conspiracy theories have white labels, non-partisan/ideological conspiracy theories have gray labels. Horizontal black bars are 95% confidence intervals.

Figure 4 displays each of the 585 correlations involved in the main analysis. While we refrain from probing these quantities individually, a brief examination of the partisan/ideological attitudes and identities can reveal instances where correlations differ directionally. For example, we observe very weak or statistically non-significant correlations for partisanship, ideology, and support for Biden and Trump when it comes to conspiracy theory beliefs that do not involve partisan/ideological considerations (those with gray labels), such as those regarding the moon landing, UFOs, assassinations (e.g., MLK, JFK, RFK), cellphones, 5G service, or the pharmaceutical industry.

We do, however, observe systematic patterns with relatively strong, statistically significant correlations when it comes to conspiracy theories involving partisan and ideological considerations (those with white labels), including those addressing Barack Obama’s birth certificate, Hillary Clinton’s supposed dealings with Russia, Republicans stealing elections, Jeffrey Epstein (a one-time Trump associate), liberal donor George Soros, and, in many cases, COVID-19 (an issue about which Donald Trump publicly proffered many conspiracy theories). These findings suggest that beliefs in conspiracy theories that accuse a contemporary partisan or ideological actor, or that are parroted by partisan or ideological media and elites, will be correlated with partisanship and ideology. Beliefs in conspiracy theories that do not accuse a partisan or ideological actor, and are not parroted by partisan or ideological elites, are unlikely to be strongly correlated with partisanship or ideology.

**Figure 5:**
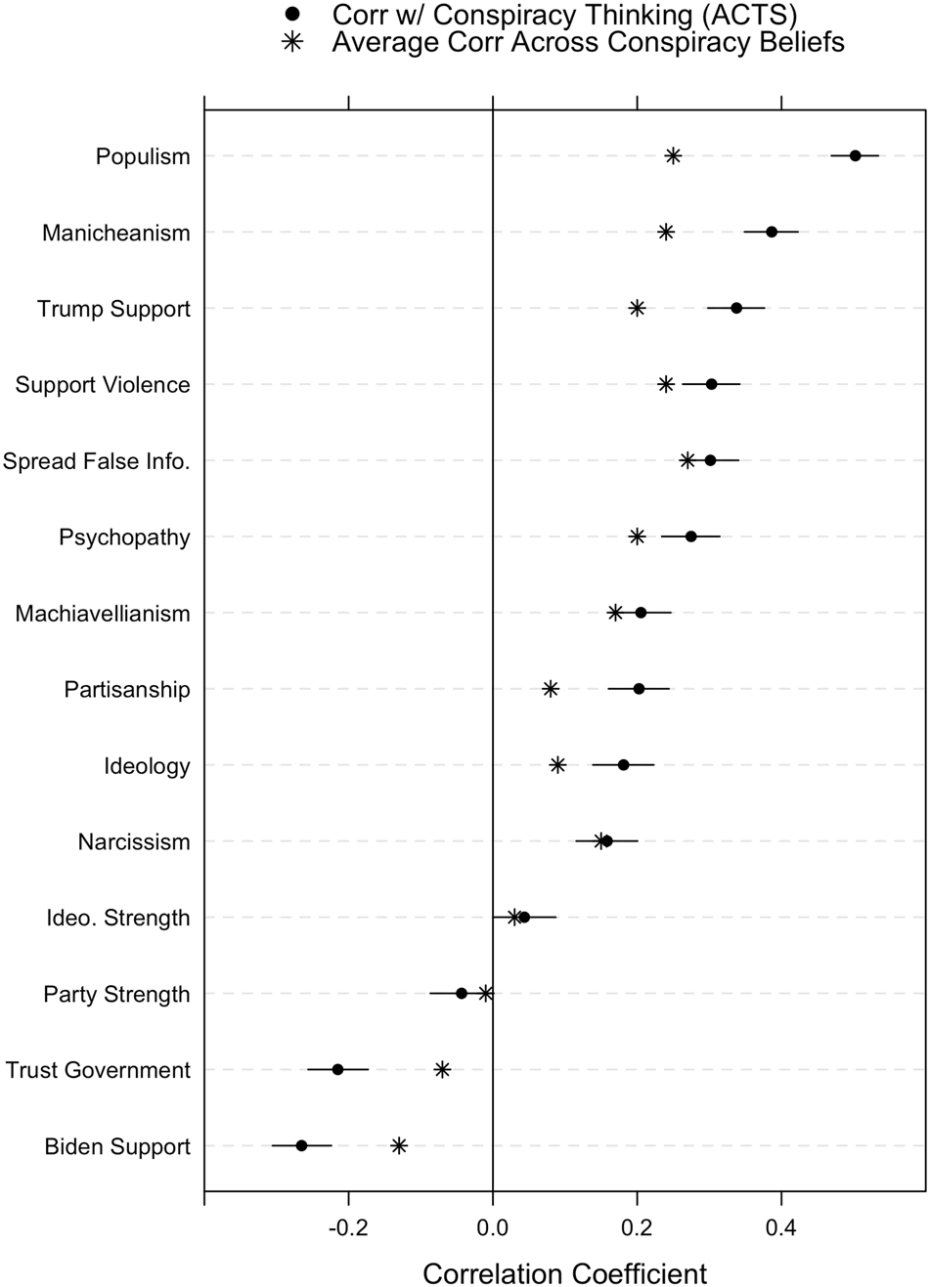
Pearson correlations between conspiracy thinking (ACTS) and each psychological and political correlated, with 95% confidence intervals. Also depicts average correlation for each correlate across all 39 conspiracy theory beliefs.

In our final analysis, we examine the correlation between each of the 15 psychological and political characteristics and the ACTS, our measure of generalized conspiracy thinking. Figure 5 displays these correlations in order of magnitude. We also overlay the average correlation between the political and psychological characteristics and all 39 specific conspiracy theories (the distribution means displayed in Fig. 1). We find that the correlations between the characteristics and the ACTS tend to mirror the average correlations across all 39 specific conspiracy theory beliefs. Indeed, the strongest, most consistent correlates of specific conspiracy theory beliefs tend to be the strongest correlates of the ACTS. In a sense, the ACTS behaves empirically like an average across many specific conspiracy theory beliefs—precisely what we should expect of (a measure of) a predisposition^60^, even though previous studies have only assumed, rather than empirically tested, this.

More specifically, we observe relatively strong, significant correlations with psychological and non-partisan/ideological political characteristics: the dark triad, sharing false information online, populism, Manicheanism, and support for political violence. There are, however, some minor discrepancies from the analysis presented in Fig. 1 (e.g., trust in government, support for political candidates). In each case where we observe a difference, the correlation with the ACTS is larger in absolute value than the average correlation across specific beliefs.

## Discussion

We investigated variability in the magnitude, direction, and statistical significance of correlations between 15 different psychological and political characteristics identified by the conspiracy theory belief literature and beliefs in 39 specific conspiracy theories, capturing a total of 585 relationships. We also interrogated the robustness of these findings across question types and utilizing a multivariate framework. Finally, we compared the correlations across specific conspiracy beliefs to those between the psychological and political characteristics we considered and conspiracy thinking, as operationalized by the ACTS.

We found that the psychological (dark triad, propensity to share false information online) and non-partisan/ideological political (populism, Manicheanism, support for political violence) traits tend to exhibit the strongest and most consistent correlations across a wide variety of beliefs in conspiracy theories. The ACTS was the strongest correlate of specific beliefs—it is reassuring, though not surprising, that it is a significant predictor of every conspiracy theory belief we examined. We also found that partisan/ideological attitudes and identities were less strongly correlated with specific conspiracy theory beliefs, on average, and exhibited a great deal of variability across beliefs. Indeed, left–right orientations such as these were most strongly correlated with beliefs in conspiracy theories that involved partisan actors and groups (e.g., Clinton, Trump, Republicans) or had ideological implications (e.g., global warming hoax beliefs). Finally, patterns in the correlations between individual characteristics we considered and conspiracy thinking (ACTS) were quite similar to patterns in the correlations with specific conspiracy theory beliefs. Where such correlations diverged, relationships with conspiracy thinking were stronger in absolute magnitude compared to the average correlations across specific conspiracy theory beliefs.

These findings have several implications for the study of conspiracy theory beliefs. First, researchers should be circumspect about generalizing from one or a small number of conspiracy theories. Not all conspiracy theories are created equal! Such an observation is reinforced when examining the partisan and ideological correlates of conspiracy theory beliefs. In particular, the basic nature of these relationships is heavily contingent on the specific conspiracy theories probed because conspiracy theories are differentially attractive depending on one’s social and political identities^61, 62^. Further, the factors associated with belief in one conspiracy theory may not speak to the general tendency to believe in conspiracy theories^60^. Nonetheless, the extant literature has focused heavily on some conspiracy theories, such as those regarding climate change^63–65^, Barack Obama’s birth certificate^12, 13, 66^, and the terror attacks of 9/11/2001^67–69^, oftentimes attempting to draw generalizable inferences about the broader tendency toward conspiracy theorizing^70^. While these conspiracy theories are, of course, important and worthy of study, our findings show that they are not representative of all conspiracy theories. Thus, generalizations based on analyses involving these conspiracy theories can be misleading if not properly qualified and contextualized.

One practical solution for those seeking to study conspiracy theories generally—as opposed to intentionally focusing on one or a few—involves utilizing a variety of conspiracy theory beliefs, ideally spanning various characteristics or dimensions, such as those identified by Brotherton et al.^46^. Another practical solution involves studying conspiracy thinking, the predisposition to believe in conspiracy theories, rather than the beliefs in specific conspiracy theories^46, 71–74^. Given that patterns in the average correlation between the psychological and political characteristics and the 39 conspiracy theory beliefs we considered tend to mirror patterns in those characteristics’ correlation with conspiracy thinking, researchers might consider employing measures of conspiracy thinking in many cases, rather than measures of beliefs in specific conspiracy theories. The ACTS and similar operationalizations (e.g., the CMQ^75^ and GCBS^46^) have the advantage of being demonstrably reliable, valid, and measurement invariant across countries and contexts. Furthermore, they are typically composed of a small number of survey items, thereby making them a robust and thrifty alternative to specific conspiracy theory beliefs. Even in instances where researchers are interested in specific beliefs, perhaps because of their potential consequences (e.g., those about vaccines, election fraud, minority groups), they might also examine conspiracy thinking as a window into how a specific belief might deviate from the average. Overall, the robust relationship between conspiracy thinking and beliefs in specific conspiracy theories demonstrated here suggests that there is still enormous potential for theory development and empirical testing when it comes to understanding the characteristics, experiences, processes, and situational factors that promote or inhibit conspiracy thinking.

Finally, our findings have implications for social and political debates and policies regarding the spread of conspiracy theories. Whereas conspiracy theories certainly pose a political problem, they may not always be a left–right partisan or ideological problem. We found positive and negative correlations with partisan and ideological identities, as well as support for Biden and Trump, signaling that those on the political left and right both believe in conspiracy theories when it is psychologically, politically, or socially expedient to do so (e.g., to reduce cognitive dissonance, bolster one’s group-image, denigrate an out-group). In such cases, measures involving correctives from co-partisans can be successful^14, 76^. However, some of the strongest and most consistent correlates of conspiracy theory beliefs involve psychological and political characteristics that are markers of anti-social, conflictual tendencies (e.g., dark triad traits, support for violence, Manicheanism). Efforts to correct or pre-bunk conspiracy theory beliefs are less likely to be successful among individuals exhibiting such traits.

We encourage researchers who are actively developing interventions aimed at dissuading people from adopting conspiracy theory beliefs to examine the effectiveness of their strategies across a large range of conspiracy theories that vary in numerous ways and to specifically consider strategies for reaching the most cantankerous—and perhaps the most dangerous—conspiracy theory believers. For example, treatments designed to “correct” conspiracy beliefs might not only include high-quality information from epistemic authority figures, which the most conspiratorial individuals are likely to ignore, but also acknowledge that even scientists and other experts occasionally get things wrong and that the process of scientific discovery is dynamic and non-linear. Such a strategy may disarm narcissists and the most distrustful members of society by acknowledging that their beliefs and worldviews have some merit, thereby making them more receptive to authoritative information. In general, more research examining the impact of debunking, prebunking, and the like—conditional on high levels of distrust, populism, Manicheanism, and dark triad personality traits—must be conducted.

### Limitations and future directions

Despite the relatively large number of correlates we employ, many other correlates that have been identified by the rapidly growing literature (e.g., criminal activity, anomie, paranoid ideation, depression) are not included in this study due to the space limitations of our survey^3, 4, 77^. We encourage future studies to replicate and expand our analyses, using additional psychological and political traits across a broad range of specific conspiracy theory beliefs to understand the robustness of the relationships observed in past work. Since they are based on cross-sectional, observational data we must also acknowledge that our analyses are incapable of shedding light on causal pathways between the psychological and political characteristics we identified and conspiracy theory beliefs. The conspiracy belief literature would benefit greatly from more studies that employ different research designs, especially those involving panel data and experimental manipulations.

We also recognize that our analysis was conducted on a U.S. sample and during a tumultuous political time when one political party was continuing to contest the outcome of a recent election. While we do not expect our focus on the U.S. to impact inferences about psychological characteristics, political dynamics do vary across sociopolitical contexts, as does the relative salience of specific conspiracy theories^21^. In other words, it could be that case that the dynamics of partisan and ideological predispositions are contingent not only on the specific conspiracy theory beliefs assessed, but also on the broader political climate (as is certainly the case for feelings toward political candidates, who differ from election to election). For example, the “conspiracy theories are for losers” hypothesis holds that those who identify with the party that loses an election or is perceived to lack power are more likely to exhibit conspiracy theory beliefs, especially about the out-party^21, 47, 78^. For these reasons, we also encourage future studies to replicate and expand our study in different countries and other time points to account for the potential impact of situational factors.

## Supplementary Information

Supplementary Information.

## Data Availability

All data and replication code is available on the Open Science Framework: https://osf.io/c6f2y/.
