# Supplementary material for: The psychological and political correlates of conspiracy theory beliefs
Source: Sci Rep. 2022 Dec 15;12:21672. doi: 10.1038/s41598-022-25617-0 (PMC9751515; doi:10.1038/s41598-022-25617-0)
Supplement: Supplementary file 1 — Supplementary Information. [file 41598_2022_25617_MOESM1_ESM.pdf]

Supplementary Information:

"The Psychological and Political Correlates of Conspiracy Theory Beliefs"

Table of Contents

I. Sociodemographic composition of sample, page 2

II. Question wording and variable coding, pages 3–6

III. Reproduction of Figure 1 by question format, pages 7–8

IV. Correlations between predictor variables, page 9

I. Sociodemographic composition of sample

Table A1: Sociodemographic information about original U.S. sample, compared to 2010 U.S. Census estimates.

Characteristic	2010 Census Estimate	Qualtrics May 2021
Age	38	48
High school degree	88	97
Some college or more	59	71
Female	51	50
White	72	62
Black	13	14
Hispanic	16	16
n		2,021

Note: All entries are percentages except age, which is the median.

While median age is slightly higher for our surveys than the 2010 Census estimates, recall that we are able to poll on only those age 18 years old and older, whereas the Census provides the median age of all Americans.

II. Question wording and variable coding

Table A2: Beliefs in 39 different conspiracy theories.

Question wording	Percent Believe
1. Do you think one man was responsible for the assassination of President Kennedy, or do you think there were others involved?*	56
2. The one percent (1%) of the richest people in the U.S. control the government and the economy for their own benefit.	52
3. Do you think the government is keeping information from the public that shows U.F.O.'s (Unidentified Flying Objects) are real or that aliens have visited the Earth?*	50
4. Jeffrey Epstein, the billionaire accused of running an elite sex trafficking ring, was murdered to cover-up the activities of his criminal network.	48
5. There is a "deep state" embedded in the government that operates in secret and without oversight.	44
6. Do you feel that the Assassination of Senator Robert Kennedy was the act of one individual or part of a larger conspiracy?*	43
7. The dangers of genetically-modified foods are being hidden from the public.	40
8. The number of deaths related to the coronavirus has been exaggerated.	36
9. The Food and Drug Administration is deliberately preventing the public from getting natural cures for cancer and other diseases because of pressure from drug companies.	35
10. Elites, from government and Hollywood, are engaged in a massive child sex trafficking racket.	34
11. Do you feel that the Assassination of Martin Luther King was the act of one individual or part of a larger conspiracy?*	33
12. Hillary Clinton conspired to provide Russia with access to nuclear materials.	29
13. The dangers of vaccines are being hidden by the medical establishment.	29
14. Coronavirus was purposely created and released by powerful people as part of a conspiracy.	29
15. Billionaire George Soros is behind a hidden plot to destabilize the American government, take control of the media, and put the world under his control.	26
16. A powerful family, the Rothschilds, through their wealth, controls governments, wars, and many countries' economies.	26
17. The coronavirus is being used to force a dangerous and unnecessary vaccine on Americans.	24
18. Do you think the U.S. government has engaged in the assassination of entertainers who have tried to spread a counterculture message they didn't like, such as John Lennon, Kurt Cobain, Tupac Shakur, and others, or not?*	20
19. Do you believe that the pharmaceutical industry is in league with the medical industry to "invent" new diseases in order to make money, or not? *	20
20. Health officials know that cell phones cause cancer but are doing nothing to stop it because large corporations won't let them.	20
21. Certain U.S. government officials planned the attacks of September 11, 2001, because they wanted the United States to go to war in the Middle East.	19

22. Barack Obama faked his citizenship to become president.	19
23. Some people have argued that President Franklin D. Roosevelt knew about Japanese plans to bomb Pearl Harbor but did nothing about it because he wanted an excuse to involve the U.S. (United States) on the side of the allies in the war. *	19
24. Do you believe media or the government adds secret mind-controlling technology to television broadcast signals, or not? *	18
25. Do you believe global warming is a hoax, or not? *	18
26. Some people are hiding the truth about the December 14, 2012 school shooting at Sandy Hook Elementary in order to advance a political agenda.	16
27. Do you think there is, or is not, a national conspiracy to kill police?*	16
28. Republicans won the presidential elections in 2016, 2004, and 2000 by stealing them.	15
29. Do you completely agree, mostly agree, mostly disagree, or completely disagree that AIDS is a form of systematic destruction of minorities like blacks and Hispanics?	15
30. Do you believe the government adds fluoride to our water supply, not for dental health reasons, but for other, more sinister reasons, or not? *	13
31. The U.S. government is mandating the switch to compact fluorescent light bulbs because such lights make people more obedient and easier to control.	12
32. The coronavirus is being used to install tracking devices inside our bodies.	12
33. Do you think that the Reagan campaign made a deal with the Iranians to hold the American hostages in Iran until after the 1980 presidential election or not? *	12
34. Bill Gates is behind the coronavirus pandemic.	11
35. Do you think there was a police conspiracy to frame O.J. Simpson or not? *	10
36. Thinking about space exploration, do you think the government staged and faked the Apollo moon landings, or don't you feel that way? *	10
37. 5G cell phone technology is responsible for the spread of the coronavirus.	7
38. Are you a believer in QANON? *	6
39. Do you believe that Osama bin Laden is dead, or do you think he is still alive?*	5

Note: Where response options are not dichotomous (e.g., yes/no, believe/don't believe), the proportion expressing belief is those who "agree" or "strongly agree" with a sentiment. *dichotomous response

Conspiracy thinking. (each item is 1=strongly disagree, 5=strongly agree; M=3.11, SD=1.00, α=0.86):

1. Much of our lives are being controlled by plots hatched in secret places.
2. Even though we live in a democracy, a few people will always run things anyway.
3. The people who really 'run' the country, are not known to the voters.
4. Big events like wars, the current recession, and the outcomes of elections are controlled by small groups of people who are working in secret against the rest of us.

Machiavellianism. Please tell us how much you agree or disagree with each of the statements below (each item is 1=strongly disagree, 5=strongly agree; M=2.09, SD=0.91, α=0.84):

1. I tend to manipulate others to get my way.
2. I have used deceit or lied to get my way.
3. I have used flattery to get my way.
4. I tend to exploit others towards my own end.

Narcissism. Please tell us how much you agree or disagree with each of the statements below (each item is 1=strongly disagree, 5=strongly agree; M=2.42, SD=0.98, α=0.87):

1. I tend to want others to admire me.
2. I tend to want others to pay attention to me.
3. I tend to seek prestige or status.
4. I tend to expect special favors from others.

Psychopathy. Please tell us how much you agree or disagree with each of the statements below (each item is 1=strongly disagree, 5=strongly agree; M=2.12, SD=0.87, α=0.81):

1. I tend to lack remorse.
2. I tend to be unconcerned with the morality of my actions.
3. I tend to be callous or insensitive.
4. I tend to be cynical.

Populism. Please tell us how much you agree or disagree with each of the statements below (each item is 1=strongly disagree, 5=strongly agree; M=3.80, SD=0.81, α=0.82):

1. Elected officials talk too much and take too little action.
2. What people call “compromise” in politics is really just selling out on one's principles.
3. Established politicians who claim to defend our interests, only take care of themselves.
4. The established elite and politicians have often betrayed the people.

Trust in government. “The federal government in Washington can be trusted to do what is right.” (M=2.62, SD=1.14)

1. Strongly disagree
2. Disagree
3. Neither agree nor disagree
4. Agree
5. Strongly agree

Share false information. “I share information on social media about politics even though I believe it may be false.” (M=1.81, SD=1.09)

1. Strongly disagree
2. Disagree
3. Neither agree nor disagree
4. Agree
5. Strongly agree

Manicheanism. "Politics is a battle between good and evil." (*M*=3.25, *SD*=1.17)

1. Strongly disagree
2. Disagree
3. Neither agree nor disagree
4. Agree
5. Strongly agree

Support violence. "Violence is sometimes an acceptable way for Americans to express their disagreement with the government." (*M*=2.16, *SD*=1.24)

1. Strongly disagree
2. Disagree
3. Neither agree nor disagree
4. Agree
5. Strongly agree

Ideology. Self-placement; 1=extremely liberal, 7=extremely conservative. (*M*=4.04, *SD*=1.80)

Partisanship. Self-placement; 1=strong Democrat, 7=strong Republican. (*M*=3.73, *SD*=2.20)

Party extremity. "Folded" partisanship measure. (*M*=1.91, *SD*=1.11)

1. True independent
2. Lean toward party
3. Weak partisan
4. Strong partisan

Ideological extremity. "Folded" ideology measure. (*M*=1.37, *SD*=1.17)

1. Moderate/centrist
2. Leaner
3. Weak identifier
4. Strong identifier

Trump support. "Feeling thermometer" ranging from 0 (very "cold," negative) to 100 (very "warm," positive). (*M*=39.70, *SD*=39.15)

Biden support. "Feeling thermometer" ranging from 0 (very "cold," negative) to 100 (very "warm," positive). (*M*=50.51, *SD*=38.12)

III. Reproduction of Figure 1 by question format

Below, we reproduce Figure 1, subsetting by general question format. In Figure A1, we present the distributions of product-moment correlations between predictors and conspiracy belief questions that employ ordinal response formats (e.g., five-point "strongly disagree" to "strongly agree"). In Figure A2, we present the distributions of point-biserial correlations between predictors and conspiracy belief questions that employ dichotomous response formats (e.g., agree vs. disagree, believe vs. do not believe). Even though these do not perfectly match each other in terms of average correlation, standard deviation, and proportion significant, the patterns are similar. It is to be expected that point-biserial correlations are weaker than product-moment. We should also note that there are more conspiracy theory questions involving partisan/ideological figures/parties/topics employing the ordinal response format than the dichotomous format. Altogether, we do not see any reason to believe that question format is systematically impacting the relative patterns we observe in Figure 1.

Figure A1: Distribution of Pearson correlation coefficients **for ordinal conspiracy belief questions**, by psychological and political correlates, across all conspiracy theory beliefs. Mean, standard deviation, and percentage of cases where correlation was statistically significant ($p<0.05$) appears in text. *P*-values corrected for multiple comparisons via Benjamini-Hochberg procedure.

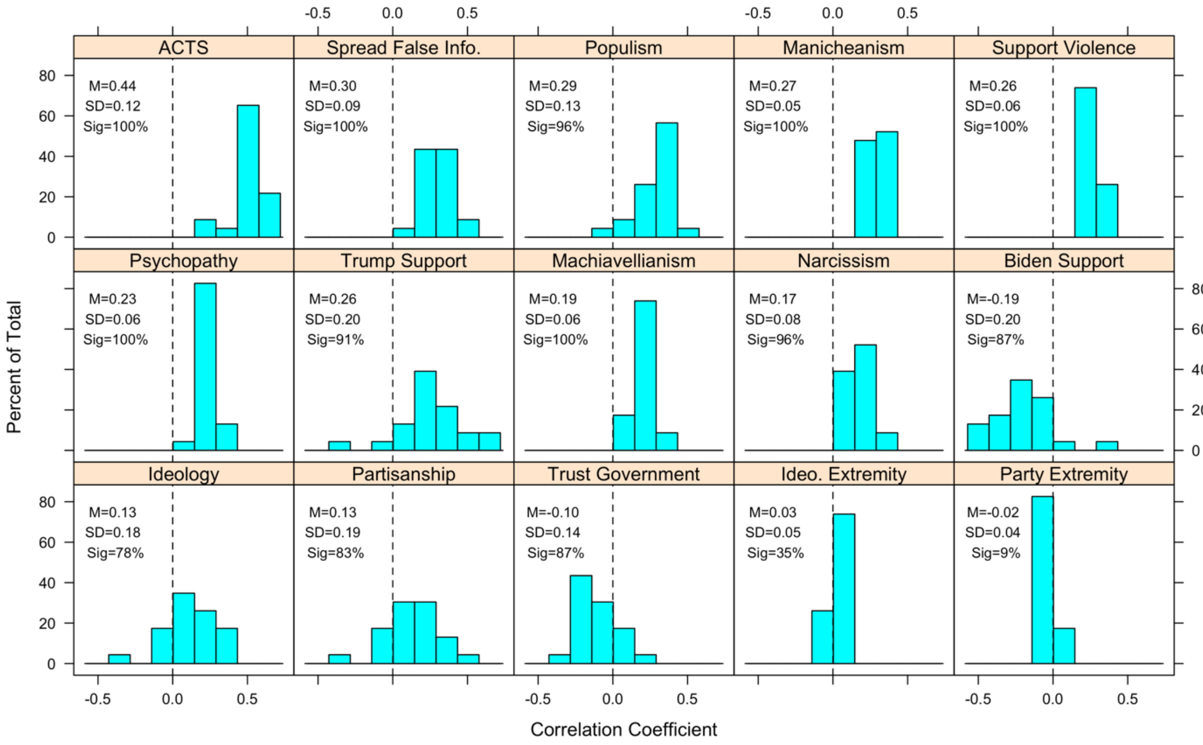

Figure A2: Distribution of point-biserial correlation coefficients **for dichotomous conspiracy belief questions**, by psychological and political correlates, across all conspiracy theory beliefs. Mean, standard deviation, and percentage of cases where correlation was statistically significant (p<0.05) appears in text. *P*-values corrected for multiple comparisons via Benjamini-Hochberg procedure.

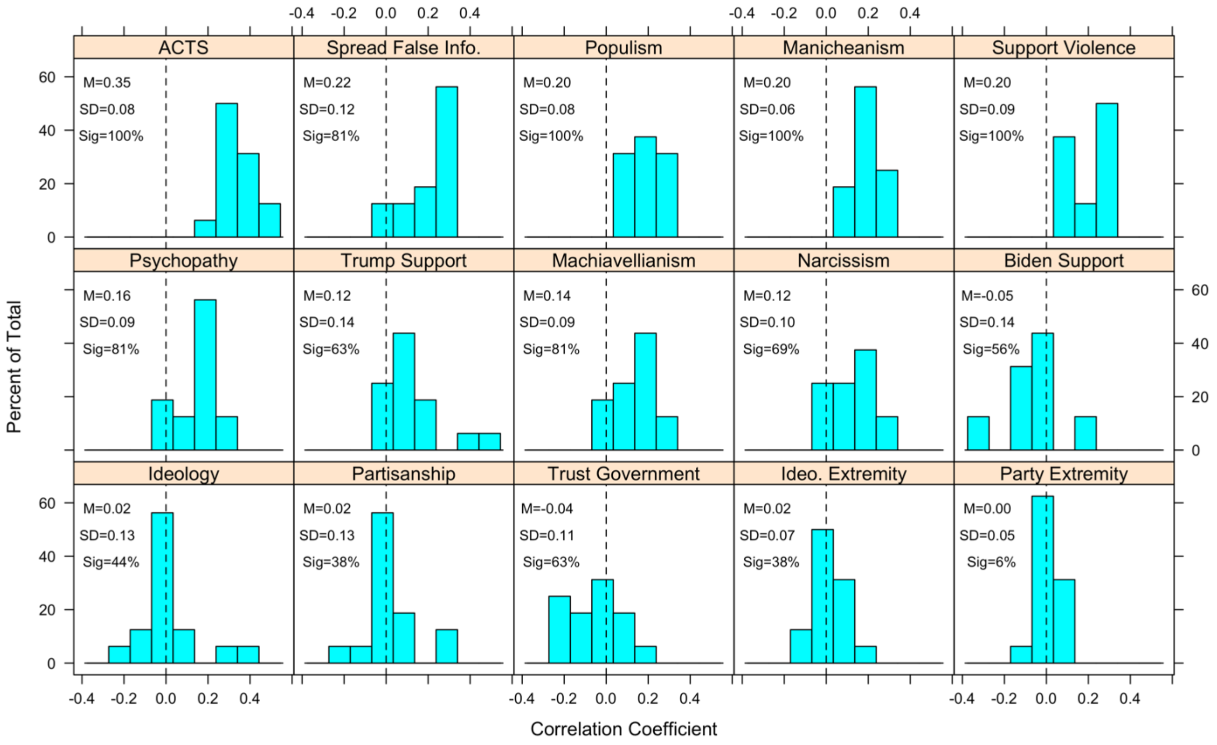

IV. Correlations between predictor variables

Table A3: Pearson correlations between predictor variables.

	1	2	3	4	5	6	7	8	9	10	11	12	13	14
1 ACTS	1.00													
2 False Info.	0.30***	1.00												
3 Populism	0.50***	0.02	1.00											
4 Manicheanism	0.39***	0.23***	0.27***	1.00										
5 Violence	0.30***	0.48***	0.13***	0.18***	1.00									
6 Trust Gov.	-0.22***	0.28***	-0.37***	-0.01	0.12***	1.00								
7 Machiavellianism	0.21***	0.38***	0.07***	0.07**	0.35***	0.14***	1.00							
8 Narcissism	0.16***	0.47***	-0.07**	0.13***	0.34***	0.30***	0.46***	1.00						
9 Psychopathy	0.27***	0.39***	0.11***	0.11***	0.36***	0.11***	0.52***	0.36***	1.00					
10 Partisanship	0.21***	-0.10***	0.24***	0.07**	-0.07**	-0.36***	-0.05*	-0.14***	-0.01	1.00				
11 Ideology	0.19***	-0.05*	0.21***	0.10***	-0.07**	-0.29***	-0.10***	-0.15***	-0.02	0.65	1.00			
12 Partisan Extremity	-0.04	0.13***	-0.03	0.12***	0.04	0.15***	0.01	0.10***	-0.02	-0.09***	-0.02	1.00		
13 Ideology Extremity	0.04	0.03	0.10***	0.10***	0.04	-0.03	0.00	0.04	0.01	0.10***	0.03	0.42***	1.00	
14 Trump Support	0.34***	0.08***	0.27***	0.24***	0.03	-0.28***	-0.01	-0.01	0.04	0.67***	0.53***	0.09***	0.21***	1.00
15 Biden Support	-0.27***	0.10***	-0.33***	-0.07**	0.02	0.48***	0.05*	0.17***	0.01	-0.70***	-0.55***	0.17	0.17***	-0.05*

Note: $^{***}p<0.001$, $^{**}p<0.01$, $^{*}p<0.005$
